# Methylphenidate and physical activity for cancer-related fatigue in immunotherapy patients: Response to letter to the editor

**DOI:** 10.1017/S147895152610279X

**Published:** 2026-06-09

**Authors:** Sriram Yennurajalingam, Eduardo Bruera

**Affiliations:** 1 Palliative Care and Rehabilitation Medicine, The University of Texas MD Anderson Cancer Center, Houston, TX, USA; 2 Department of Palliative Care & Rehabilitation Medicine, The University of Texas MD Anderson Cancer Center, Houston, TX, USA

Response to Letter to the Editor:

We thank the Editor for the opportunity to respond to the comments outlined in the Letter to the Editor. Our detailed responses are provided as follows.

1. 1:1 vs 1:2 randomization randomized clinical trials for treatment of cancer-related fatigue (CRF): Based on prior trials demonstrating individual efficacy of methylphenidate and physical activity for CRF, we prespecified a 1:2 randomization for this pilot, exploratory study (Yennurajalingam et al. 2026). This design maximized intervention exposure, improved recruitment feasibility, and optimized data yield within an unfunded study, while allowing assessment of the preliminary efficacy of the combined intervention in a population at high risk for attrition.
2. The intent of the 2-week physical activity intervention was to pilot test the combination of methylphenidate with a standardized physical activity regimen to improve CRF over a short, pragmatic timeframe. Determination of the optimal dose and duration of both methylphenidate and physical activity will be addressed in future, adequately powered randomized controlled trials.
3. As a pilot study, the inclusion of multiple secondary outcome measures was intentional to assess the sensitivity to change of fatigue and related domains across several validated instruments. This approach was designed to inform the selection of the most responsive and clinically meaningful measures for future adequately powered fatigue trials for CRF in patients receiving PD-1 immunotherapy.
4. Heterogeneous population and lack of generalizability: This pilot study aimed to explore the efficacy of interventions for CRF in patients receiving PD-1 immunotherapy. Key confounders were minimized through strict eligibility criteria, thereby limiting concerns related to the most important confounders related to CRF; assessment of efficacy across specific cancer subgroups and broader racial and ethnic representation will be addressed in a planned larger phase III study.

In summary, our findings suggest that the combination of methylphenidate plus physical activity is very promising, and further research is justified.

Best regards,

Sriram Yennurajalingam

Eduardo Bruera

## References

[ref1] Yennurajalingam S, Fellman B, Williams L, et al. (2026) Managing fatigue with methylphenidate and physical activity during cancer immunotherapy treatment. *Palliative and Supportive Care* 24, e1. 10.1017/S1478951525101375 PMC13166623 41423710

